# Analyses of spike protein from first deposited sequences of SARS-CoV2 from West Bengal, India

**DOI:** 10.12688/f1000research.23805.1

**Published:** 2020-05-18

**Authors:** Feroza Begum, Debica Mukherjee, Dluya Thagriki, Sandeepan Das, Prem Prakash Tripathi, Arup Kumar Banerjee, Upasana Ray

**Affiliations:** 1 CSIR-Indian Institute of Chemical Biology, Kolkata, West Bengal, 700032, India; 2 North Bengal Medical College and Hospital, Siliguri, West Bengal, 734012, India; 3 Department of Biochemistry, North Bengal Medical College and Hospital, Sushrutanagar, West Bengal, 734012, India

**Keywords:** Spike, India, West Bengal, Mutations

## Abstract

India has recently started sequencing SARS-CoV2 genome from clinical isolates. Currently only few sequences are available from three states in India. Kerala was the first state to deposit complete sequence from two isolates followed by one from Gujarat. On April 27, 2020, the first five sequences from the state of West Bengal (Eastern India) were deposited on GISAID, a global initiative for sharing avian flu data. In this study, we have analysed the spike protein sequences from all five isolates and also compared their similarities or differences with other sequences reported in India and with isolates of Wuhan origin. We report one unique mutation at position 723 and another at 1124 in the S2 domain of spike protein of the isolates from West Bengal only.  There was one mutation downstream of the receptor binding domain at position 614 in S1 domain which was common with the sequence from Gujarat (a state of western India).  Mutation in the S2 domain showed changes in the secondary structure of the spike protein at region of the mutation. We also studied molecular dynamics using normal mode analyses and found that this mutation decreases the flexibility of S2 domain.  Since both S1 and S2 are important in receptor binding followed by entry in the host cells, such mutations may define the affinity or avidity of receptor binding.

## Introduction

The SARS-CoV2 (a member of Coronaviruses) outbreak occurred in Wuhan, China in the year 2019, and was recently declared a pandemic, which has affected countries worldwide. To design antiviral therapeutics/vaccines it is important to understand the genetic sequence, structure and function of the viral proteins. When a virus tries to adapt to a new environment, in a new host, in a new geographical location and a new population, it may make changes to its genetic make-up which in turn bring slight modifications in viral proteins. Such variations would help the virus to utilize the host’s machinery to best in favour virus survival and propagation. Since the host’s immune system eventually learns to identify an infecting pathogen and starts producing protective antibodies, a virus often changes its structural proteins so that it escapes the host’s immune system and continues to infect the host cells. Coronaviruses have been long known to undergo rapid mutations in its RNA genome ^1^. Such mutations are reflected in changes in the amino acid sequences of its structural and non-structural proteins.

The spike protein is a structural protein of SARS-CoV2 that forms a homotrimer on the surface of the vital lipid envelope ^2^. This trimer is made up of monomers consisting of S1 and S2 subunits. S1 subunit helps in attachment to the host cell receptor, while S2 subunit helps in fusion to host cell and entry. Thus, the spike protein has been an area of interest for designing vaccine and antiviral candidates against SARS-CoV2. Since the spike protein tends to mutate, it is important to obtain a broad mutation profile of this protein from extensive genome sequencing from different geographical locations of the world. Targeting areas of the spike protein that do not undergo mutation, i.e. conserved regions, would be the key to designing effective broad spectrum antivirals or vaccine.

## Methods

### Sequences

We downloaded the five new SARS-CoV2 sequences from West Bengal (EPI_ISL_430468; EPI_ISL_430467; EPI_ISL_430465; EPI_ISL_430464; EPI_ISL_430466) from the GISAID database under the EpiCov icon by specifying location as India and the spike protein sequences corresponding to Kerala isolates (QIA98583 and QHS34546) ^3^, Gujarat isolate (QJC1949.1) ^4^ and Wuhan isolate (QIS29982.1) from the NCBI virus database. Wuhan isolate was used as the original SARS-CoV-2 sequence for sequence comparisons. For this study we have considered only the complete sequences (>29,000bp) with high coverage.

For the West Bengal isolates, the nucleotide sequences corresponding to the spike protein were selected and then translated on ExPASY Translate tool to obtain the protein sequences.

### Sequence alignments and structure

All the spike protein sequences were aligned using the multiple sequence alignment platform of CLUSTAL Omega. The alignment file was viewed using MView and differences in the sequence or the amino acid changes were recorded.

CFSSP (Chou and Fasman secondary structure prediction) server was used to predict secondary structures of SARS-CoV2 spike protein.

To study the effect of mutation on the conformation, stability and flexibility of the spike protein, the SARS-CoV-2 structure was downloaded from RCSB PDB. We used the available SARS-CoV-2 spike ectodomain structure (open state) (PDB ID: 6VYB). The 6VYB structure was uploaded on DynaMut software (University of Melbourne, Australia) ^5^ and changes in vibrational entropy and the atomic fluctuations and deformation energies due to mutation were determined. For atomic fluctuation and deformation energy calculations, calculations were performed by the software over first ten non-trivial modes of the molecule.

## Results and discussion

The first set of five sequencing data from clinical isolates of SARS-CoV2 from the state of West Bengal, India was submitted on 27/2/2020 by the National Institute of Biomedical Genomics (NIBMG) in collaboration with ICMR-National Institute of Cholera and Enteric Diseases (ICMR-NICED). The sequences were submitted to the GISAID database.

We downloaded all the sequences from West Bengal ( Table 1) and performed a nucleotide translation to obtain respective spike protein sequences. All these spike protein sequences were first aligned in CLUSTAL Omega to check for similarities or differences. We found that at position 723, all the isolates from West Bengal except EPI_ISL_430466 had ‘T’ (other isolates had ‘I’). Also, at position 1124, two of the isolates (EPI_ISL_430468 and EPI_ISL_430464) showed mutation from ‘G’ to ‘V’. We used spike protein sequence translated from one of these sequences (EPI_ISL_430468) as representative of SARS-CoV2 spike from West Bengal for our further comparison with other states.

**Table 1.** Accession numbers of the sequences from West Bengal used for the analyses with specific regions of sample collection.

Virus name	Accession number	Region of collection within West Bengal	Gender, Age (years)
hCoV-19/India/S2/2020	EPI_ISL_430468	Kolkata	Male, 48
hCoV-19/India/S11/2020	EPI_ISL_430467	East Medinipur	Male, 25
hCoV-19/India/S5/2020	EPI_ISL_430465	Darjeeling	Female, 44
hCoV-19/India/S3/2020	EPI_ISL_430464	Kolkata	Male, 20
hCoV-19/India/S6/2020	EPI_ISL_430465	Tehatta	Male, 11

**Figure 1.**
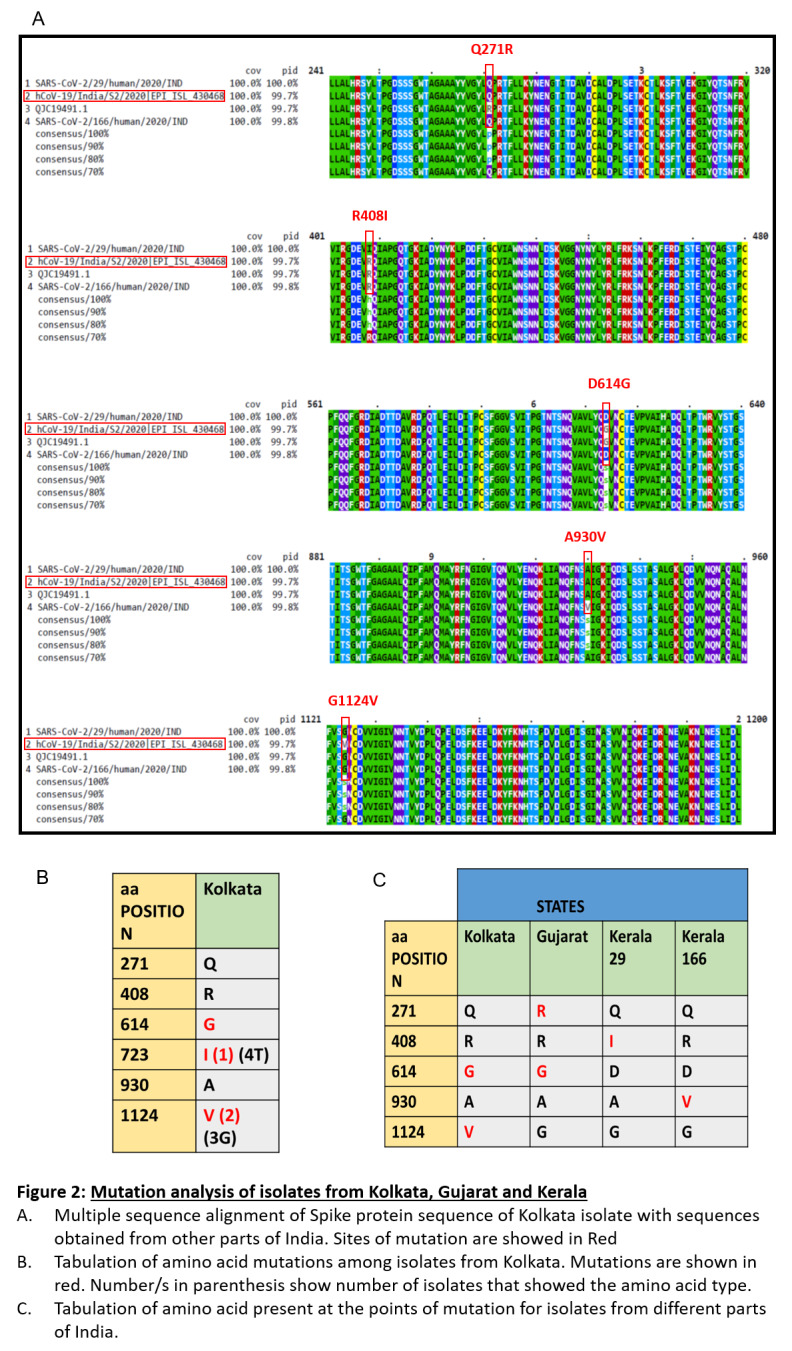
Mutation analysis of isolates from Kolkata, Gujarat and Kerala. ( **A**) Multiple sequence alignment of Spike protein sequence of Kolkata isolate with sequences obtained from other parts of India. Sites of mutation are showed in Red. ( **B**) Tabulation of amino acid mutations among isolates from kolkata. Mutations are shown in red. Number/s in parenthesis show number of osolates that showed the amino acid type. ( **C**) Tabulation of amino acid present at the points of mutation for isolates from different parts of india.

Since, we had access to sequences for SARS-CoV2 only from three states in India (Kerala, Gujarat and now West Bengal), we compared all those sequences to detect possible changes ( Figure 1). We considered the original Wuhan sequence as the wild type for comparison. We found six different amino acid positions that were mutated in these isolates overall. We had recently published the details with respect to the spike protein mutations in Kerala ^3^ and Gujarat isolates ^4^.

Here, we report that in West Bengal isolates there were three mutations in the spike protein. One of these mutations was D614G in the S1 domain. This lies near the receptor bending domain at a downstream position. Another mutation, T723I lie further downstream in the S protein in the S2 domain. Mutation G1124V is the third mutation and lie in the S2 domain. While D614G was also found in the isolate from Gujarat but not in Kerala isolates, T723I and G1124V mutation were exclusively found in the isolates of West Bengal. None of the isolates from other parts of India had this mutation.

Although T723I was also exclusive for West Bengal, it appeared only in one isolate. Thus, we characterized mutation G1124V which appeared in two of the isolates. Both glycine (G) and valine (V) are non-polar amino acids with aliphatic R groups. Glycine has no side chain whereas valine is bulkier due to its side chain. A change from glycine to valine can thus potentially disrupt the local folding of the protein. For example, it was shown that G to V change in a P-glycoprotein changed its drug specificities ^6^.

**Figure 2.**
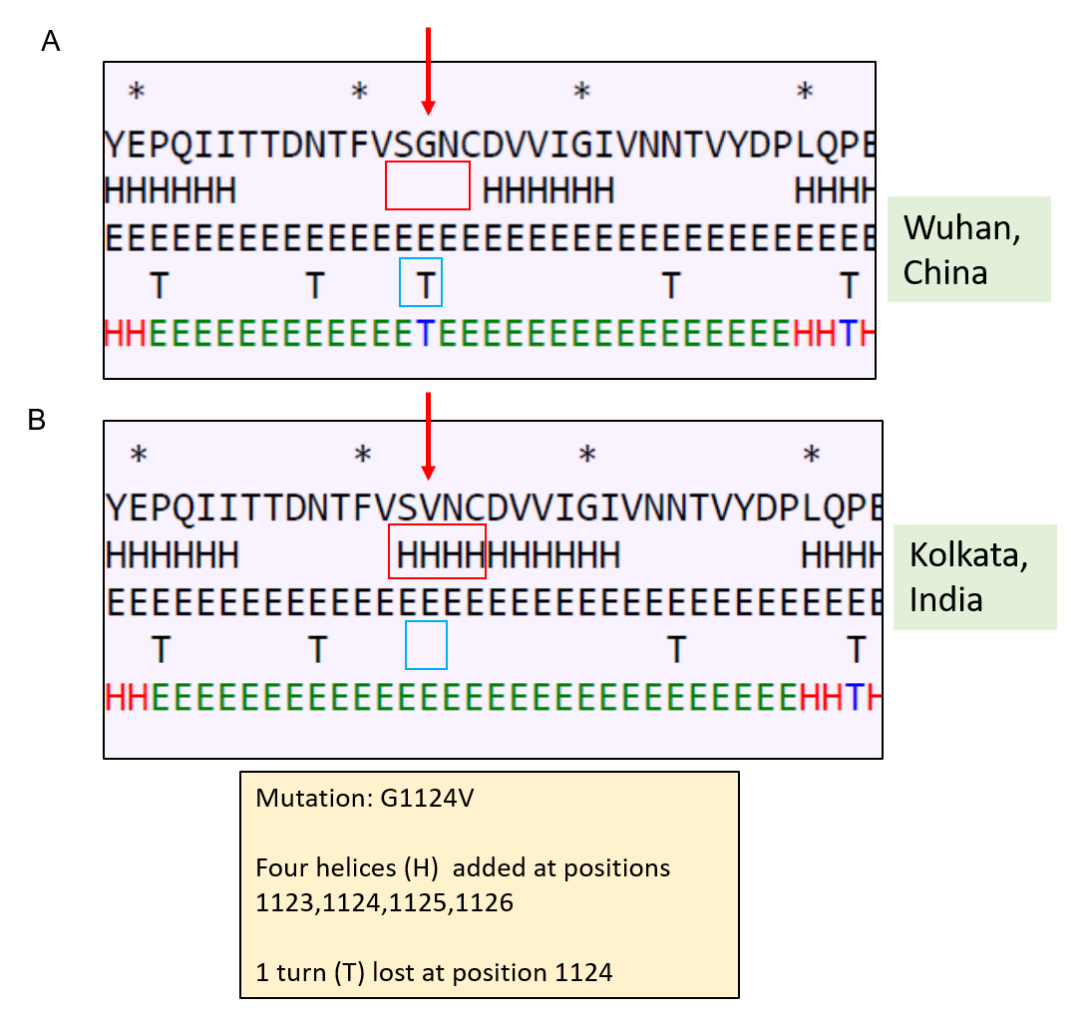
Effect of mutation at position 1124 on secondary structure of Spike protein. ( **A**) Secondary structure of Spike protein of Wuhan isolate (the area around the residue 1124 has been shown); ( **B**) secondary structure of Spike protein of Kolkata isolate showing effect of mutation on the secondary structure.

Secondary structure prediction showed changes in and around the site of mutation ( Figure 2). In the mutant spike there was a loss of turn structure from position 1124 and addition of four helices at positions 1123, 1124, 1125 and 1126. This change in secondary structure might lead to change in function of S2. S2 helps in fusion process of the spike protein and thus mutation in S2 may have altered receptor spike interactions and thus infectivity.

**Figure 3.**
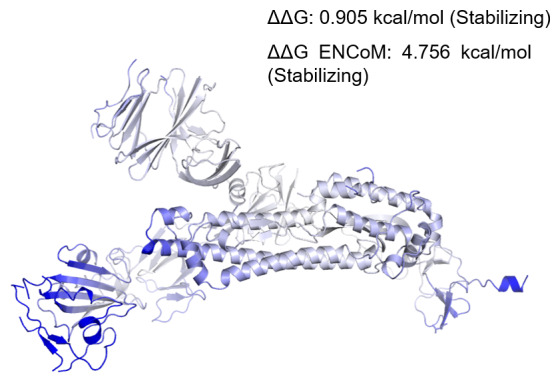
Δ Vibrational Entropy Energy Between Wild-Type and Mutant. Δ Vibrational Entropy Energy Between Wild-Type and Mutant ΔΔSVib ENCoM: -4.445 kcal.mol-1.K-1. Amino acids were coloured as per the vibrational entropy change due to mutation. Blue represents rigidification of structure.

**Figure 4.**
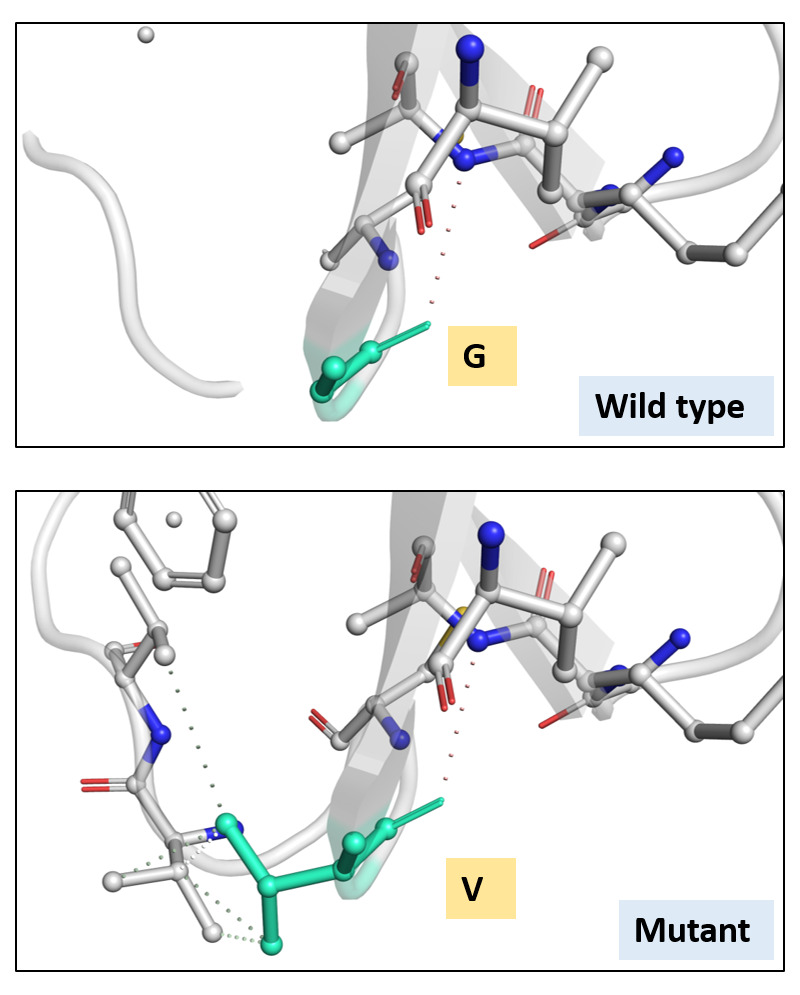
Interatomic interaction. Wild-type and mutant residues are coloured in light-green and are also represented as sticks alongside with the surrounding residues which are involved on any type of interactions.

To correlate if changes in secondary structure are also reflected in the dynamics of the protein in its tertiary structure, we performed normal mode analyses and studied protein stability and flexibility. Change in vibrational entropy energy (ΔΔSVib ENCoM) between the wild type Wuhan isolate and the West Bengal isolate was -4.445 kcal.mol ^-1^.K ^-1^ ( Figure 3). The ΔΔG was 0.905 kcal/mol and the ΔΔG ENCoM was 4.756 kcal/mol. All these suggested a stabilizing mutation in this type of spike. The interatomic interactions have been shown in Figure 4.

**Figure 5.**
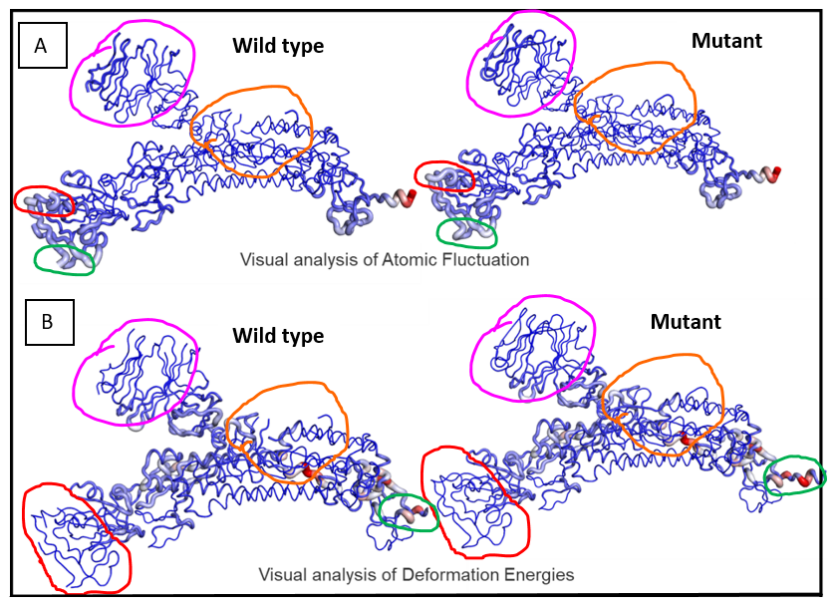
Visual analysis of fluctuations and deformation energies. Magnitude of ( **A**) atomic fluctuation and ( **B**) deformation has been shown using thin to thick lines coloured blue (low), white (moderate) and red (high).

Analyses of atomic fluctuations and deformation energies showed visible changes ( Figure 5). Atomic fluctuations calculate the measure of absolute atomic motion whereas deformation energies detect the measure of flexibility of a protein. Figure 5 shows the visual representations of the atomic fluctuation and deformation energies where positions that could be visibly detected to be different have been marked.

This is the first report of mutations of such types in the isolates of the state of West Bengal and further sequencing followed by sequence analyses would help expanding the knowledge about variations of spike protein in human SARS-CoV. These variations might lead to virus diversification and eventual emergence of variants, antibody escape mutants, strains and serotypes. Also, mutations might help the virus to expand its tissue tropism and adjust with the host environment better. Therefore, elaborate studies on sequence variations should be done, which would in turn help in better therapeutic targeting.

## Data availability

### Underlying data

Accession numbers for sequences used in this study can be found in Table 1 and the test. GSAID is a free-to-use resource, but requires registration prior to accessing.

### Extended data

Figshare: Figure F1000corrected.pdf, https://doi.org/10.6084/m9.figshare.12254033.v2 ^7^.

This project contains the following extended data:

- Images raw images as created on DynaMut software:
  ○ Deformation_protein_158809623989_wild_type
  ○ Deformation_protein_G943V_158809623989_mutant_1124
  ○ Diff_A_G943V_158809623989_Kolkata_1124_mutation_6VYB23805-V1-1-fluctuation_protein_158809623989wild_type
  ○ Fluctuation_protein_G943V_158809623989_mutant_1124
  ○ Protein_cleanedcontacts_A_943_158809623989_wild_type
  ○ Protein_G943Vcontacts_A_943_158809623989_Mutant_Kolkata_1124

## References

[1] Wu F Zhao S Yu B: A new coronavirus associated with human respiratory disease in China. *Nature.* 2020;579(7798):265–269. 10.1038/s41586-020-2008-3 32015508 PMC7094943

[2] Tai W He L Zhang X: Characterization of the receptor-binding domain (RBD) of 2019 novel coronavirus: implication for development of RBD protein as a viral attachment inhibitor and vaccine. *Cell Mol Immunol.* 2020. 10.1038/s41423-020-0400-4 32203189 PMC7091888

[3] Saha P Banerjee AK Tripathi PP: A virus that has gone viral: Amino acid mutation in S protein of Indian isolate of Coronavirus COVID-19 might impact receptor binding and thus infectivity. *BioRxiv.* 2020 10.1101/2020.04.07.029132 PMC7225408 32378705

[4] Banerjee AK Begum F Thagriki D: Novel Mutations in the S1 domain of COVID 19 Spike Protein of Isolate from Gujarat Origin, Western India. *Preprints.* 2020 10.20944/preprints202004.0450.v1

[5] Rodrigues CH Pires DE Ascher DB: DynaMut: predicting the impact of mutations on protein conformation, flexibility and stability. *Nucleic Acids Res.* 2018;46(W1):W350–W355. 10.1093/nar/gky300 29718330 PMC6031064

[6] Choi KH Chen CJ Kriegler M: An altered pattern of cross-resistance in multidrug-resistant human cells results from spontaneous mutations in the mdr1 (P-glycoprotein) gene. *Cell.* 1988;53(4):519–29. 10.1016/0092-8674(88)90568-5 2897240

[7] Ray U: Figure F1000corrected.pdf. *figshare.*Figure.2020 10.6084/m9.figshare.12254033.v2

