# Supplementary material for: Analyses of spike protein from first deposited sequences of SARS-CoV2 from West Bengal, India
Source: F1000Res. 2023 Sep 27;9:371. Originally published 2020 May 18. [Version 2] doi: 10.12688/f1000research.23805.2 (PMC7309413; doi:10.12688/f1000research.23805.2)
Supplement: Peer review history

# Reviewer report: Reviewer response for version 1

**DOI:** 10.5256/f1000research.26268.r63543

**Published:** 2020-06-18

**Authors:** Nicolas Cuburu

**Affiliations:** 1 Laboratory of Cellular Oncology, National Cancer Institute, National Institutes of Health, Bethesda, MD, USA

This article from Begum *et al.* entitled “Analyses of spike protein from first deposited sequences of SARS-CoV2 from West Bengal, India" reports a detailed analysis spike protein sequences deposited in GSIAD repository from clinical isolates of SARS-CoV2. The report relies on algorithms to predict the secondary structure of the SARS-CoV2 spike protein variant from Bengal and proposes that novel mutations in the SARS-CoV2 S2 domain could result in changes in the virus infectivity and escape mutants.

 Because the structures were all based on the CFSSP algorithm it would be important to show how these structures relate to the known crystal structure of SARS-CoV2 spike from Wuhan. Also, the authors compared the sequences to Wuhan's original virus, but more mutations in S2 notably have been reported in clinical isolates from other parts of the world. An important question is whether the mutations from Bengal are new and unique or whether they are found also in the circulating virus in North America and Europe for instance.

 Overall the observation and the hypothesis are interesting but at this point, it is purely speculative and these mutations could be passengers with no consequence on virus natural infection and transmission. These questions could only be solved by infection experiments *in vitro* or in animal models.

 The authors have used the CFSSP method, but many other algorithms are available for secondary structure prediction. What was the rationale for choosing this specific algorithm? Would other algorithms yield similar predictions?

 In the introduction, the authors mention that the CoV undergo rapid mutations but compared to other RNA viruses CoV tends to mutate more slowly due to the proofreading exonuclease.

 Figure 1: it is not clear what consensus 100% 90% etc… represent.

 Minor comments: few typos (osolate instead of isolate etc…)

Is the work clearly and accurately presented and does it cite the current literature?

Partly

If applicable, is the statistical analysis and its interpretation appropriate?

Not applicable

Are all the source data underlying the results available to ensure full reproducibility?

Yes

Is the study design appropriate and is the work technically sound?

Yes

Are the conclusions drawn adequately supported by the results?

Partly

Are sufficient details of methods and analysis provided to allow replication by others?

Yes

Reviewer Expertise:

I have limited expertise on computational approaches for protein structure prediction

I confirm that I have read this submission and believe that I have an appropriate level of expertise to confirm that it is of an acceptable scientific standard.

# Reviewer report: Reviewer response for version 1

**DOI:** 10.5256/f1000research.26268.r63544

**Published:** 2020-06-02

**Authors:** Jalaj Gupta

**Affiliations:** 1 Stem Cell Research Center, Department of Hematology, Georg-Speyer-Haus, Lucknow, Uttar Pradesh, India

Begum *et al.* submitted the manuscript ‘Analyses of spike protein from first deposited sequences of SARS-CoV2 from West Bengal, India’ where COVID19 virus sequences retrieved from GISAID database have been studied for possible mutations. Authors report three mutations out of which two are unique (T723I and G1124V) and one shared (D614G) with other geographical locations. This is a timely study, unrevealing two unique mutations that can be useful for further studies on the behavior of COVID19. However, the authors did not clarify why they characterized only one mutant but not the other.

1) The two mutations (T723I and G1124V) discussed in the manuscript should be similarly presented. Authors have described detailed analysis of G1124V mutation while T723I mutation is not discussed in detail. Therefore entropy calculations, interatomic interactions, deformation energy, and atomic fluctuation studies should also be done for mutation T723I. 
What is the effect of T723I mutation on the secondary structure? This analysis should also be done and included in Figure 3. This will further improve the quality of the manuscript.
Authors claim that the two mutations (T723I and G1124V) are unique in the West Bengal region of India. Are they prevalent elsewhere in the World as well? Authors should expand their analysis and include more COVID19 isolates from different continents/countries.

**Minor comments:**

1. The accession number of Gujarat isolate in method and figure 1 do not match. Correction needed.
2. Accession number EPI_ISL_430465  in Table 1 is duplicated. One should be EPI_ISL_430466.
3. Parameters for calculation of vibrational entropy, atomic fluctuations, and deformation energies should be described in more detail.

Is the work clearly and accurately presented and does it cite the current literature?

Yes

If applicable, is the statistical analysis and its interpretation appropriate?

Not applicable

Are all the source data underlying the results available to ensure full reproducibility?

Yes

Is the study design appropriate and is the work technically sound?

Yes

Are the conclusions drawn adequately supported by the results?

Yes

Are sufficient details of methods and analysis provided to allow replication by others?

Partly

Reviewer Expertise:

Host-pathogen interaction, Biochemical characterization of mutant versions of proteins, Cancer biology using organoid and mouse models

I confirm that I have read this submission and believe that I have an appropriate level of expertise to confirm that it is of an acceptable scientific standard, however I have significant reservations, as outlined above.

# Reviewer report: Reviewer response for version 1

**DOI:** 10.5256/f1000research.26268.r63545

**Published:** 2020-05-26

**Authors:** Pawan Kumar Maurya

**Affiliations:** 1 Department of Biochemistry, Central University of Haryana, Mahendragarh, Haryana, India

The manuscript submitted reports mutations in newly sequenced SARS-CoV-2 isolates of West Bengal origin. The information presented here is a useful addition to the ongoing COVID 19 research. While the manuscript is well written and sufficiently documented, the authors should consider adding the following additional information. 

  

1. Mutation 614 D to G has been in discussion in recent times extensively. The authors could add few lines in the discussion about the possible importance of this mutation as they also report the same in West Bengal’s sequences.
2. DynaMut analysis for only G1124V has been done. Since mutation at the 723 position is also unique, a similar analysis for the same should also be included.

Is the work clearly and accurately presented and does it cite the current literature?

Yes

If applicable, is the statistical analysis and its interpretation appropriate?

Yes

Are all the source data underlying the results available to ensure full reproducibility?

Yes

Is the study design appropriate and is the work technically sound?

Yes

Are the conclusions drawn adequately supported by the results?

Yes

Are sufficient details of methods and analysis provided to allow replication by others?

Yes

Reviewer Expertise:

Clinical Biochemistry

I confirm that I have read this submission and believe that I have an appropriate level of expertise to confirm that it is of an acceptable scientific standard.
